# The Effect of Varying Abutment Heights on Stress Distribution in Different Bone Densities: A Finite Element Analysis Study

**DOI:** 10.3390/ma18194561

**Published:** 2025-09-30

**Authors:** Mario Ceddia, Tea Romasco, Giulia Marchioli, Alessandro Cipollina, Luca Comuzzi, Adriano Piattelli, Natalia Di Pietro, Bartolomeo Trentadue

**Affiliations:** 1 Department of Mechanics, Mathematics and Management, Polytechnic University of Bari, 70125 Bari, Italy; bartolomeo.trentadue@poliba.it; 2 Department of Medical, Oral and Biotechnological Sciences, “G. D’Annunzio” University of Chieti-Pescara, 66100 Chieti, Italy; tea.romasco@unich.it (T.R.); giulia.marchioli912@gmail.com (G.M.); natalia.dipietro@unich.it (N.D.P.); 3 Independent Researcher, 92019 Sciacca, Italy; alexandros1960@libero.it; 4 School of Dentistry, Saint Camillus International University of Health and Medical Sciences, 00131 Rome, Italy; apiattelli51@gmail.com; 5 Independent Researcher, 31020 San Vendemiano, Italy; luca.comuzzi@gmail.com; 6 Facultad de Medicina, UCAM Universidad Católica San Antonio de Murcia, 30107 Murcia, Spain

**Keywords:** finite element analysis (FEA), transgingival height, abutment height, stress distribution, bone density, implant biomechanics, dental implants

## Abstract

The biomechanical performance of dental implants is affected by both abutment height and bone quality, which influence stress distribution around the implant and the preservation of surrounding bone. This study used three-dimensional finite element analysis (FEA) to assess the combined effects of these factors. Two implants with abutment heights of 3 mm and 6 mm were modeled and placed in mandibular bone blocks representing class II and class IV bone, according to Lekholm and Zarb’s classification. A static load of 150 N, inclined at 6° buccolingually, was applied during the analysis. The simulation results showed that increasing the abutment height raises stress on the implant, leading to greater stress transfer to the peri-implant bone. The von Mises stress levels were higher in the crestal cortical bone of the class IV model with a 6 mm abutment (126 MPa). Notably, peak stresses exceeding 300 MPa were localized at the implant-abutment connection. These findings suggest that abutment height is a critical factor that negatively affects the biomechanical response, especially in low-density bone, although longer abutments offer biological benefits. This highlights the importance of minimizing the crown-to-implant ratio to reduce overload, preserve bone, and prevent mechanical failure complications.

## 1. Introduction

Today, oral implantology is one of the most reliable options for treating partial and complete edentulism, thanks to advances in biological understanding, materials, and surgical and prosthetic techniques [1,2,3]. While the long-term survival rates of dental implants are generally high, treatment success cannot be judged solely by the longevity of implants in the arch. Functional, aesthetic, and biological factors must also be considered [4,5]. Therefore, maintaining marginal bone levels and ensuring the stability of peri-implant soft tissues are especially important for a positive long-term outlook [6,7,8].

From a biomechanical perspective, implant placement initiates the process of transferring masticatory loads from the prosthetic components to the surrounding bone. Unlike natural teeth, which have a periodontal ligament that cushions occlusal forces and distributes stress across a broad surface, implants are firmly anchored in the bone and lack an elastic absorption mechanism [9,10,11]. Consequently, stress distribution depends solely on direct bone-to-implant contact (BIC), proper osseointegration, and the design of the prosthetic reconstruction [12,13,14,15,16]. This means that unfavorable distribution can cause stress concentrations and micro-deformations in the bone, potentially leading to clinical issues such as marginal bone resorption or early implant failure [11]. Implant failures are generally classified as either mechanical or biological [17]. Mechanical failures include component fracture, screw loosening, and excessive micro-movements under load, which can hinder healing or damage the bone-implant interface [18,19].

The peri-implant soft tissues also play a vital role in maintaining the health of the surrounding implant areas. The mucosal seal around the abutment is essential as a biological barrier against bacterial invasion, which can lead to peri-implantitis and marginal bone loss (MBL) over time [20]. The stability and quality of the soft tissues depend on patient-related factors, such as gingival thickness, oral hygiene, smoking, implant design, and the type of implant-abutment connection [21,22]. Furthermore, clinical studies have shown that the amount of bone resorption may be influenced by factors like surgical trauma, the position of the micro-gap, implant geometry, inter-implant distance, and patient habits [23,24,25].

The importance of abutment height in preserving peri-implant tissues has recently received more attention. Several studies indicate that longer abutments (>2 mm) are associated with less MBL than shorter ones, regardless of mucosal thickness [26,27]. Using longer abutments helps create a stable biological width. However, other studies suggest an upper limit of 4 mm, as very long abutments can increase the crown-to-implant ratio and the lever arm, which can raise stresses on the implant connection and marginal bone [28,29,30]. It has been observed that increasing abutment height by 1 mm can cause a 20% increase in bone stress [31].

Another factor interacting with abutment height is bone density. High-density bone provides a more even load distribution, whereas low-density bone is more vulnerable to stress concentrations, especially in thin cortical areas, which increases the risk of resorption and implant failure [32,33,34]. However, the combined effect of abutment height and bone density on biomechanical behavior remains unclear.

In modern engineering, finite element analysis (FEA) is a widely used tool for understanding how abutment height influences biomechanical performance. This numerical simulation technique is typically employed to develop and assess three-dimensional (3D) models of the bone-implant-prosthesis system under various loading conditions [35,36]. Assuming rigid contact between bone and implant, FEA can accurately evaluate force distribution, micro-deformations, and areas where stress may concentrate. The ability to modify parameters such as implant geometry, cortical thickness, and abutment height makes this method especially valuable for simulating complex clinical scenarios and predicting long-term results [37,38].

The present study aimed to use FEA to examine how different abutment heights affect stress distribution in the peri-implant bone and at the implant-abutment connection. This is important because existing evidence in the literature is conflicting, and there are no clear guidelines. The study also evaluated bone density, focusing on classes II and IV according to Lekholm and Zarb’s classification. The goal was to identify the optimal abutment height that promotes favorable load distribution, preserves peri-implant tissues, and minimizes the risk of biological and biomechanical complications. This research helped establish predictive parameters for the clinical selection of the definitive abutment.

Furthermore, the study aimed to thoroughly explore the long-term effects of different implant combinations, particularly focusing on the mechanical stability of the connection and the risk of fracture. Understanding the stress concentration mechanisms in the implant-abutment connection is critical, as this area is one of the most common sites for biomechanical complications.

## 2. Materials and Methods

### 2.1. Modeling

**Figure 1:**
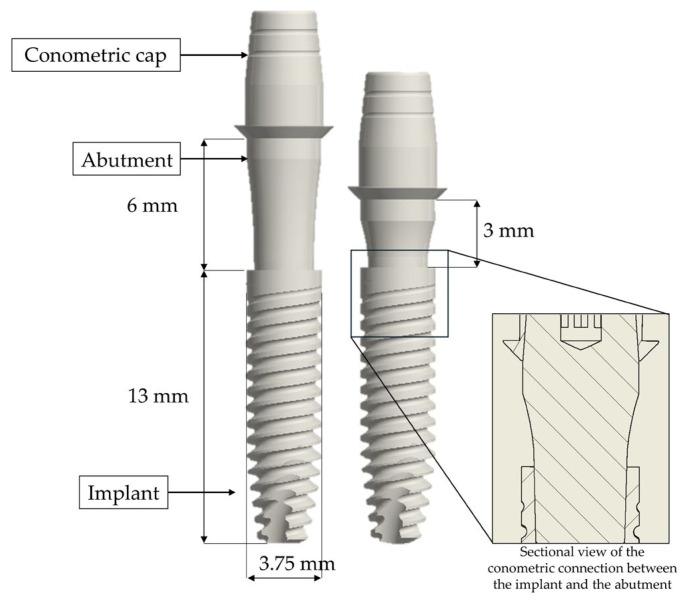
Schematic illustration of the implant-abutment assembly. The models feature an implant (13 mm × 3.75 mm; AoN Implants Srl, Grisignano di Zocco, Italy) connected to abutments with heights of 3 mm and 6 mm. Inset: sectional view of the conometric connection (Morse-taper connection) between the implant and the abutment.

The study examined two prosthetic models, both characterized using the same dental implant (13 mm × 3.75 mm; AoN Implants Srl, Grisignano di Zocco, Italy). These implants feature a flat apex design aimed at minimizing trauma during bone insertion, improving placement accuracy, and enhancing primary stability [39]. A notable feature of these implants is the internal conometric connection (Morse-taper connection), used to connect the implant and the abutment. This type of connection provides an exact fit, effectively limiting micro-movements during flexural loads [40] (Figure 1).

**Figure 2:**
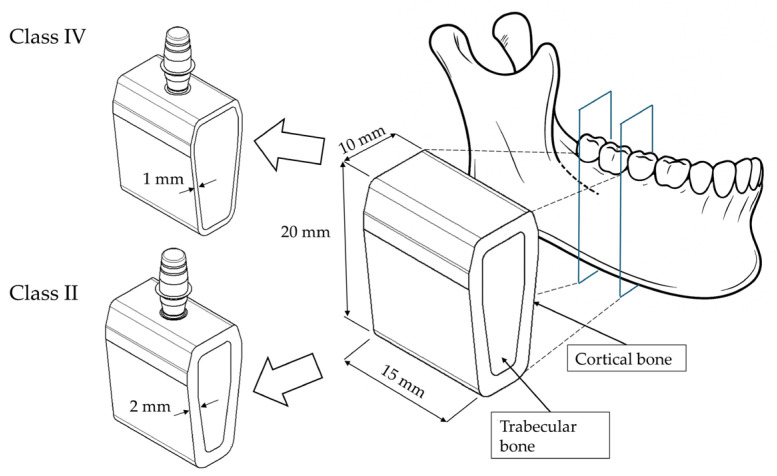
Three-dimensional (3D) modeling of the bone block extracted from a section of a mandible.

Moreover, the Morse-taper connection helps evenly distribute masticatory forces to the surrounding bone, reducing excessive stresses on the peri-implant tissues [41,42]. In this study, the implants were paired with abutments of 3 mm and 6 mm heights. These sizes were chosen because they match the minimum and maximum abutment heights offered by the manufacturer, representing the clinically relevant range of options. Three-dimensional models of the implants and prosthetic components were created using computer-aided design (CAD) software (Autodesk Inventor 2024.3, San Francisco, CA, USA) and used for the analyses. Using the same software, a bone block was also modeled from a cross-sectional image of a computed tomography (CT) scan of an edentulous posterior mandible (Figure 2).

For the analysis, two bone qualities were assumed based on the classification of Lekholm and Zarb (classes II and IV) [43]. Class II bone was modeled with a cortical thickness of 2 mm, while class IV bone was assigned a cortical thickness of 1 mm along the buccal and lingual surfaces. The overall dimensions of the posterior mandibular bone segment were 20 mm in vertical height, 15 mm in mesiodistal width, and 10 mm in buccolingual width at the alveolar crest. Finally, the various components were assembled by positioning the implant at the crestal level and creating an osteotomy with a diameter smaller than that of the implant, ensuring optimal adaptation between the bone and the implant surface.

### 2.2. Materials

After modeling the components, it was necessary to define the mechanical properties of the materials being studied. Bone exhibits anisotropic mechanical behavior, with elastic properties varying along the three spatial directions [44,45,46]. However, a reasonable approximation can be made by assuming that the elastic moduli of cortical bone in the buccolingual and inferosuperior directions are not significantly different. Similarly, the elastic moduli of trabecular bone in the buccolingual and mesiodistal directions can be considered comparable [47,48].

Based on these observations, it is reasonable to assume that bone can be modeled as a transversely isotropic material. This approach simplifies its properties to just five independent elastic parameters, compared to the nine required for an orthotropic material or the 21 required for a fully anisotropic one [49]. Additionally, assuming transverse isotropy provides a more accurate representation of bone anisotropy than the more common isotropic approximation used in other studies, which assumes identical properties in all directions.

The elastic properties were assigned based on the bone quality classification of Lekholm and Zarb (classes II and IV) (Table 1) [50,51].

Regarding the prosthetic components, a Ti-6Al-4V titanium alloy was selected for the implants because it has isotropic and homogeneous mechanical properties (Table 2) [52]. Additionally, this alloy combines high mechanical strength and corrosion resistance with excellent biocompatibility, enabling predictable osseointegration and long-term clinical stability.

### 2.3. Constraints and Loading Conditions

**Figure 3:**
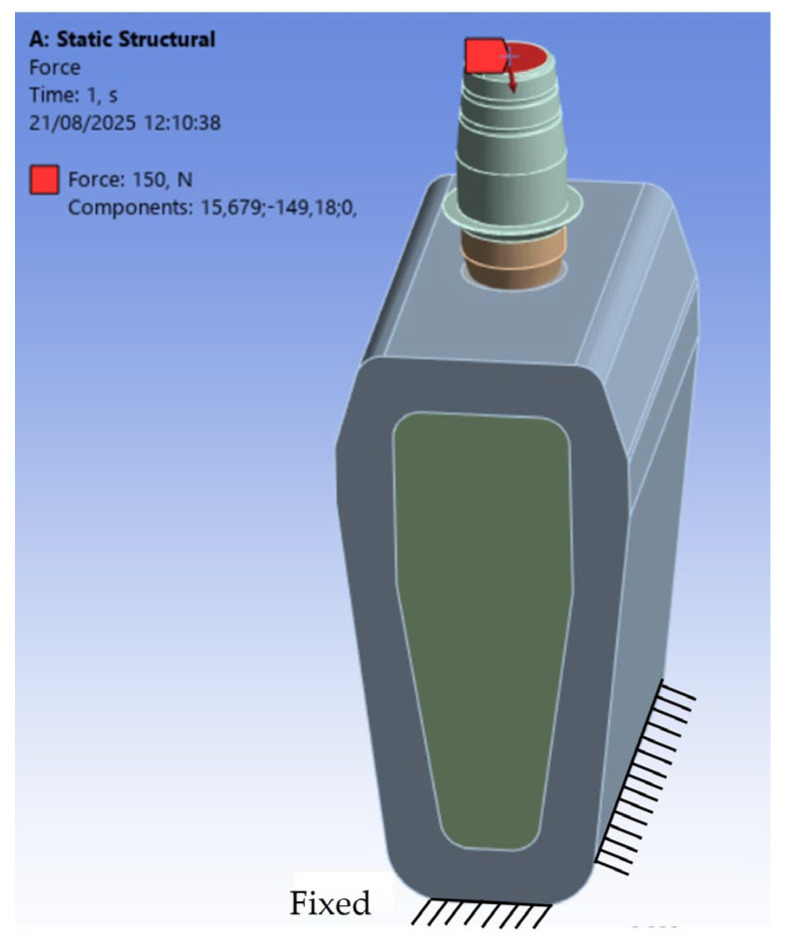
Boundary and loading conditions of the finite element analysis (FEA) model.

For this study, a static load of 150 N was applied to the superior surface of the abutment, with a 6° inclination relative to the implant’s axial direction and oriented from lingual to buccal. This loading condition was chosen to mimic the functional stresses typically acting on a second premolar or a first molar, based on the findings of Watanabe et al. [53]. To ensure the numerical model’s stability, all lateral surfaces of the bone block were constrained with zero-displacement conditions, preventing movement in the three Cartesian directions (x, y, z). The contact between the implant and bone was modeled as a bonded contact, simulating complete osseointegration with no micro-movements at the interface. Conversely, the contacts between the implant and abutment, as well as between the abutment and the crown, were defined as frictional contacts with a static coefficient of friction of 0.3 [54,55,56,57]. This setup allowed for a realistic simulation of the prosthetic connections’ biomechanical behavior under occlusal loading. Figure 3 illustrates the loading and boundary conditions used in the analysis.

### 2.4. Finite Element Analysis (FEA)

**Figure 4:**
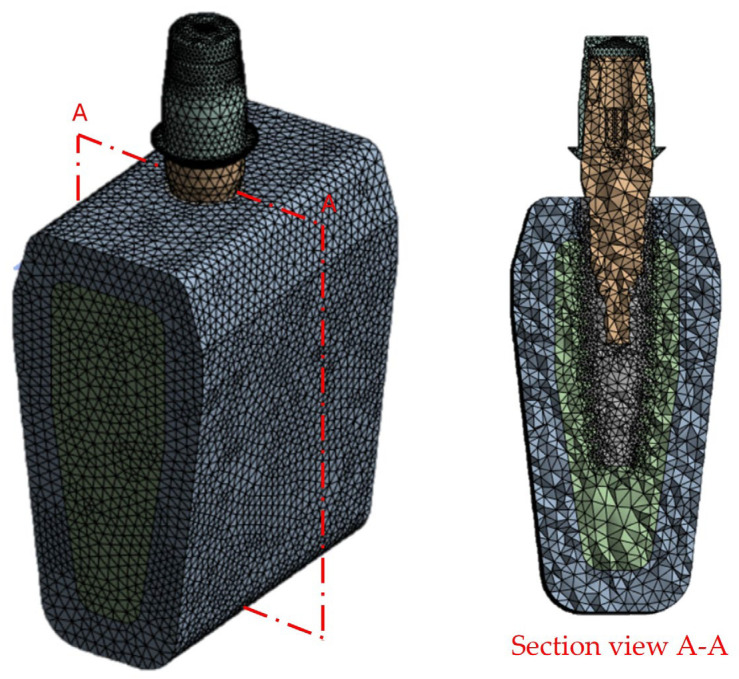
Discretized model using tetrahedral elements measuring 0.5 mm.

The FEA was conducted using ANSYS Workbench 2023 (R23^®^, ANSYS Inc., Canonsburg, PA, USA). The analysis was performed on a workstation running Windows 10 64-bit, with an Intel Core i7 processor (2.90 GHz) and 16 GB of RAM. The models were discretized with quadratic tetrahedral elements (SOLID187), which are particularly suitable for complex geometries and are frequently used in biomechanical applications to ensure accurate stress distribution. A mesh sensitivity analysis identified the optimal element size at 0.5 mm, ensuring a maximum error of 2%. The final mesh consisted of 97,123 elements and 176,563 nodes across all 3D models (Figure 4).

After implementing the 3D model in the FEA software (ANSYS Workbench 2023, R23^®^, ANSYS Inc., Canonsburg, PA, USA), the von Mises stress values and their distributions were analyzed using color maps for visual representation. The von Mises criterion was chosen for its compatibility with FEA software, effectively illustrating the mechanical behavior of components while accounting for stresses in all three spatial directions. This criterion is based on the idea that material deformation occurs when the combined stress from external forces surpasses the maximum stress the material can withstand.

## 3. Results

The color maps displayed the von Mises stress distribution in the bone and implant models. The scale bar showed the stress range (MPa), with blue indicating lower and red indicating higher values. Because the scale is software-defined, similar colors may represent different absolute stresses. Red areas correspond to regions of maximum concentration, while blue areas indicate minimal stress loading.

The FEA showed that von Mises stress was mainly focused in the crestal cortical bone, regardless of bone quality or abutment height. Trabecular bone experienced lower stresses but had greater involvement in low-density models, with stresses spreading apically. Peak values were consistently observed near the implant neck, confirming this area as critical for load transfer.

The stress concentration in the cortical bone can be explained by its material properties. Cortical bone is denser and stiffer than trabecular bone, with an elastic modulus of about 10–20 GPa. It is located in the crestal region, where occlusal loads are first transferred from the implant. Because of its rigidity, cortical bone resists deformation and therefore accumulates higher stress. In contrast, trabecular bone has a much lower modulus (0.1–2 GPa), making it more compliant. As a result, it distributes stresses over a wider area but at lower magnitudes. Cortical bone thus acts as the main load-bearing structure, while trabecular bone plays a secondary role. Only in poor-quality bone (class IV) does the trabecular component become more involved, since the thin cortical shell cannot dissipate loads on its own.

**Figure 5:**
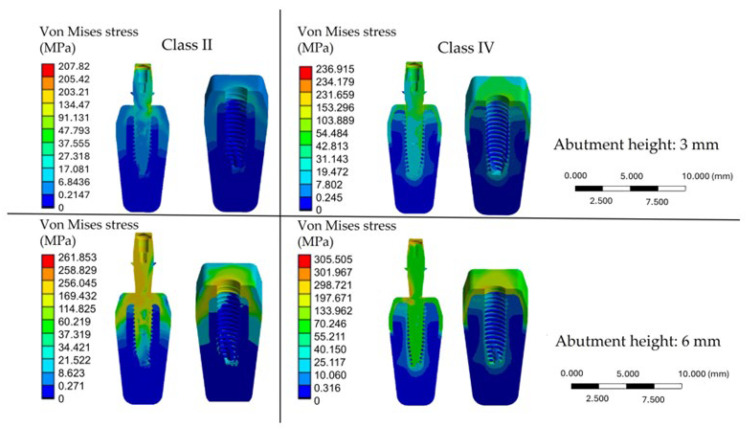
Distribution of von Mises stresses (MPa) in the bone block based on bone quality (class II and class IV) and abutment height (3 mm and 6 mm).

As shown in Figure 5, the distribution of von Mises stress was affected by both bone quality and abutment height.

In the class II bone with a 3 mm abutment, the maximum stress reached approximately 207.82 MPa, primarily concentrated in the crestal cortical bone (18.42 MPa). In contrast, the trabecular bone experienced much lower stress levels (2–3 MPa). In the class IV bone model with the same abutment height, peak stresses increased to 236.91 MPa, with the cortical bone exposed to significantly higher stresses (43.57 MPa) and greater involvement of the trabecular bone (5–11 MPa), indicating a reduced capacity to dissipate loads. These values are clinically relevant, since trabecular bone is known to tolerate stresses only up to about 10–20 MPa before micro-damage occurs, while cortical bone can withstand higher levels but may be at risk of overload when stresses exceed 100–120 MPa. The increase in abutment height, however, had the most significant impact: with a 6 mm abutment, maximum stress rose to 261.85 MPa in class II bone, accompanied by a substantial increase in crestal cortical stress (106 MPa). The most critical condition was observed in class IV bone with a 6 mm abutment, where stress reached 305.51 MPa, affecting both cortical bone (126 MPa) and trabecular bone (36.5 MPa). These values are close to or above the reported tolerance limits of both bone types, suggesting a higher risk of biological overload, particularly in low-density bone.

**Figure 6:**
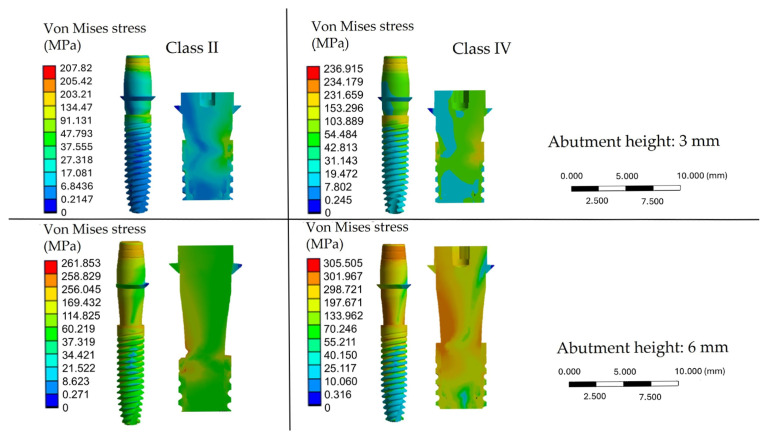
Distribution of von Mises stresses (MPa) in the implants based on bone quality (class II and class IV) and abutment height (3 mm and 6 mm).

Regarding the implant components, the FEA revealed that von Mises stress was especially concentrated at the implant-abutment connection, which represents the most mechanically critical part of the system (Figure 6).

An increase in abutment height resulted in significantly higher stress at this junction. Specifically, stresses in this area reached approximately 246 MPa with a 6 mm abutment in class II bone, whereas peak stresses exceeded 300 MPa in class IV bone. Considering that the fatigue strength of Ti-6Al-4V alloy under oral conditions may be compromised when cyclic stresses approach 250–300 MPa, these findings highlight the risk of material fatigue, abutment fracture, or screw loosening under unfavorable biomechanical conditions.

## 4. Discussion

This study employed FEA to simulate implant insertion with abutments of varying heights into low-density (class IV) and high-density (class II) bone using 3D models. The primary objective was to analyze stress distribution in the implant system based on the von Mises criterion, focusing on 3 mm and 6 mm abutments placed at the crestal level. Many systematic reviews and meta-analyses confirm the validity of using FEA to study implant biomechanics in scenarios that are hard to evaluate in vivo [58,59,60,61]. To simplify the model and facilitate the analysis, some assumptions were made, such as material homogeneity and linear elastic behavior. For the bone, a transversely isotropic model was used to more accurately reflect its anisotropic properties, in line with experimental data in the literature [62].

The simulation results demonstrated that increasing the height of the abutment elevates stress on the implant, resulting in more stress being transferred to the peri-implant bone. The crestal area of the cortical bone was identified as the region experiencing the highest stress in all conditions, regardless of bone density or abutment height. However, in class IV bone, there was an increasing involvement of the trabecular bone, with stresses spreading more extensively in the apical direction. This indicates a reduced load-bearing capacity [62]. These results are consistent with those reported by Kurniawan et al. [63], who showed that high-density bone exhibits greater mechanical strength and allows for more effective stress distribution. Conversely, low-density bone exhibits higher stress concentrations and a smaller biomechanical safety margin. Increasing the abutment height was confirmed as the most significant factor in worsening the mechanical response. With a 6 mm abutment in class II bone, maximum stresses reached 261.85 MPa; in contrast, in class IV bone, peaks over 305 MPa were observed. This phenomenon is due to the lever effect, where the larger distance between the load application point and the implant fulcrum increases stress on the mechanical structure joint.

From a clinical perspective, this evidence is particularly significant because multiple studies have identified abutment height as a key factor influencing MBL [64,65]. Retrospective analyses have shown that longer abutments help preserve bone by establishing a more stable biological width and moving the implant-prosthesis connection away from the crestal bone [66,67,68]. However, this study confirmed that although longer abutments offer a biological benefit, they also lead to a significant increase in mechanical stress, especially in cases of poor bone quality. This raises the risk of resorption due to overload. As demonstrated by Beltrán-Guijarro et al. [54], increasing abutment height increases the stress transferred to the bone and results in greater deformation of all components of the prosthesis-implant-bone complex system.

Additionally, bone quality is an essential factor in determining response. Stresses are better contained in class II bone, while critical concentrations occurred in class IV bone, especially in cortical bone. This increases the potential risk of implant failure. Yoon et al. [69] conducted a study using FEA to evaluate how bone quality (normal versus low density) and peri-implant resorption affect stress distribution within the implant-bone system. The results clearly showed that poor bone quality leads to greater vulnerability to stress concentrations, particularly in models with up to 2 mm of crestal resorption. Kurtuluş et al. [70] conducted an FEA study to assess the effect of bone density and cortical thickness on stress distribution. Their findings indicated that decreases in trabecular tissue density and reduced cortical thickness significantly increase stress on both the bone and implants. Notably, stress levels were higher in models with D4-type (low-density) bone than in denser bone.

Another key finding involves the localization of stress at the implant-abutment connection, which is confirmed as the system’s most mechanically weak point. In this area, stress levels were notably higher than in other regions, exceeding 300 MPa in class IV bone with a 6 mm abutment. Clinically, this is important because the connection is the area most prone to mechanical issues such as fractures, screw loosening, and micro-movements, which jeopardize the stability of the prosthetic joint. As Matsuoka et al. [71] observed, the highest stress levels are indeed concentrated in the connection zone, reaffirming its mechanical weakness. A systematic review by Fernández Asián et al. [72] also highlighted that the design of the implant-abutment connection significantly affects the fatigue resistance of the implant. Although the internal conometric connection reduces the risk of micro-movements compared to other designs, the elevated stress levels seen in this area indicate increased susceptibility to fatigue phenomena and potential fractures, as also reported by Lorusso et al. [73].

Therefore, these numerical results align with prior in vitro and in vivo research. Experimental examinations of implant-abutment assemblies have verified that the mechanical junction constitutes the most stressed region, with cyclic loading inducing micro-gaps and component fatigue, thereby corroborating the stress concentration observed in the present models [74]. Similarly, recent FEA and mechanical validation studies have demonstrated that cortical bone consistently bears higher stress levels than trabecular bone, especially in cases of diminished cortical thickness. This supports the current observation that the crestal cortical layer plays a crucial role in load transfer [75]. Moreover, animal studies have indicated greater BIC and stress localization within the cortical region, underscoring its significance in the mechanical stability and biological integration of the implant system [76].

In this study, some simplifications were introduced in the finite element model, which represented essential limitations for the interpretation of the results. The condition of complete osseointegration between bone and implant was assumed, without considering possible situations of partial or compromised integration, which have been shown in the literature to influence stress distribution significantly [77,78]. Moreover, the mechanical properties of bone and prosthetic materials were modeled as linear, elastic, and homogeneous, except for bone, which was considered transversely isotropic. This assumption constitutes a simplification, as several studies have highlighted the importance of incorporating nonlinear and time-dependent behaviors for more realistic simulations [79,80]. Another limitation concerns the application of static and constant loads, which do not reflect the dynamic, cyclic, and multidirectional nature of actual masticatory forces, nor the potential fatigue effects. Indeed, recent studies have demonstrated the role of bone remodeling and fatigue loading on implant performance, underscoring the need to incorporate these aspects into advanced models [81,82]. However, the use of these simplifications primarily aims to reduce computational time and costs, offering the advantage of easier parameterization and broader applicability [83].

Based on these findings, some practical clinical guidance can be proposed. In sites with poor bone quality (class IV), shorter abutments should be preferred to minimize mechanical overload and reduce the risk of MBL. In contrast, in high-density bone (class II), slightly greater abutment heights may be acceptable when required for prosthetic reasons, provided that the crown-to-implant ratio is carefully controlled. Overall, limiting excessive lever arms is essential to reduce stress concentrations at the crestal cortical bone and implant-abutment connection. It is also important to emphasize the biomechanical significance of the stress observed. Stress exceeding 100–120 MPa in cortical bone or 10–20 MPa in trabecular bone may surpass the biological tolerance of these tissues, promoting micro-damage and resorption under repeated functional loading. Likewise, peak values above 250–300 MPa at the implant-abutment connection approach the fatigue thresholds of Ti-6Al-4V alloy, potentially compromising long-term mechanical stability through screw loosening, abutment fracture, or connection wear. These aspects highlight that, beyond stress distribution trends, the absolute magnitudes reached in unfavorable conditions (long abutments in low-density bone) may have direct biological and mechanical consequences that clinicians should carefully consider in treatment planning.

## 5. Conclusions

Considering the inherent limitations of the numerical method used to evaluate stress distribution in prosthetic components and peri-implant tissues, it can be concluded that the stress transmitted to the bone-implant system depends on the abutment height in implant-supported prostheses. Specifically, increasing the abutment height results in higher stress transfer throughout the system. Additionally, bone quality plays a significant role in stress distribution; for example, lower bone density (class IV bone) tends to concentrate more stress within the cortical bone. Clinically, while longer abutments may help preserve peri-implant bone health, they can also increase mechanical loads on the bone. In cases of low-density bone, these increased loads could weaken the bone’s resistance and jeopardize the stability of the surrounding tissue.

Future research is necessary to confirm these numerical results through preclinical and clinical studies. The current analysis will also be expanded to include different implant designs, loading conditions, and long-term bone remodeling simulations. This will offer more comprehensive guidelines for clinical decision-making.

## Figures and Tables

**Table 1:** Properties of materials for transversely isotropic bones.

Properties (MPa)	High-Density Trabecular Bone (Class II)	Low-Density Trabecular Bone (Class IV)	Cortical Bone
E_x_^1^	1148	230	12,600
E_y_	210	42	12,600
E_z_	1148	230	19,400
$v_{xy}$ ^2^	0.05	0.05	0.300
$v_{xz}$	0.32	0.32	0.253
$v_{yz}$	0.01	0.01	0.253
G_xy_^3^	17	14	4850
G_xz_	108.50	87	5700
G_yz_	17	14	5700

^1^ E: Elastic or Young’s Modulus; ^2^ v : Poisson’s Ratio; ^3^ G: Shear Modulus.

**Table 2:** Properties of the materials used for the implant.

Model	Material	Young’s Modulus (GPa)	Poisson’s Ratio (*ν*)
Implant	Ti-6Al-4V	110	0.35

## Data Availability

The original contributions presented in this study are included in the article. Further inquiries can be directed to the corresponding author.

## Abbreviations

The following abbreviations are used in this manuscript:

BIC	Bone-implant contact
MBL	Marginal bone loss
FEA	Finite element analysis
3D	Three-dimensional
CAD	Computer-aided design
CT	Computed tomography
